# Racial and ethnic disparities in the incidence, healthcare utilization, and outcomes of retained placenta among delivery hospitalizations in the United States, 2016–2019

**DOI:** 10.1186/s12884-023-06097-0

**Published:** 2023-11-11

**Authors:** Wen Jiang, Wei Chen, Dong Li

**Affiliations:** 1 Department of Obstetrics and Gynecology, West China Second University Hospital, Sichuan University, Chengdu, China; 2 Key Laboratory of Birth Defects and Related Diseases of Women and Children (Sichuan University), Ministry of Education, Chengdu, Sichuan China; 3 West China Biomedical Big Data Center, West China Hospital, Sichuan University, Chengdu, China; 4 Department of Emergency, Harbor-UCLA Medical Center, 1000 W Carson St, Torrance, CA 90502 USA

**Keywords:** Disparities, Healthcare utilization, Mortality, Race/Ethnicity, Retained placenta

## Abstract

**Background:**

Retained placenta is a concern during labor and delivery. However, recent data regarding the profiles of retained placenta are scarce, especially nationwide and in minority populations. This study aimed to investigate the recent incidence of retained placenta and its associated outcomes.

**Methods:**

We retrospectively analyzed an American population-based data from the National Inpatient Sample (NIS) 2016–2019. The outcomes of interest included the incidence of retained placenta, in-hospital mortality, length of hospital stay, and hospitalization costs. We estimated the incidence for retained placenta overall and by racial and ethnic subgroups, utilizing survey weights standardized for each subgroup. Multivariable linear or logistic regression models were employed in our study to investigate the associations between retained placenta and the impact of in-hospital mortality, duration of stay, and hospitalization expenditures for the entire population and further stratified by race and ethnicity, adjusting for potential confounders.

**Results:**

Of the 13,848,131 deliveries, there were 108,035 (or 0.78%) birthing persons were identified as having retained placentas. Over time, the incidence of retained placenta increased from 730 per 100,000 (0.73%) in 2016 to 856 per 100,000 (0.86%) in 2019. Native American mothers have the highest rate of retained placenta, with a prevalence almost twice that of the general population, reaching 1,434 cases per 100,000 (1.43%). After adjusting for confounding factors, Native American mothers were more likely to have retained placenta (odds ratio [OR], 1.56; 95% confidence interval [CI], 1.35–1.81), whereas Black (OR, 0.92; 95% CI, 0.88–0.97) and Hispanic mothers (OR, 0.84; 95% CI, 0.80–0.89) were significantly less likely to have retained placenta than White mothers. Furthermore, those who delivered with a retained placenta were significantly associated with higher in-hospital mortality, a longer duration of stay, and hospitalization expenditures, which were disproportionately varied by maternal race and ethnicity.

**Conclusions:**

The incidence of retained placenta among people undergoing vaginal delivery is exhibiting an upward trend over time, with notable variations observed across different ethnic groups by unclear mechanisms. The ramifications of these findings have the potential to impact the clinical management of maternal health care and the creation of health policies, specifically in relation to the Native American birth population.

**Supplementary Information:**

The online version contains supplementary material available at 10.1186/s12884-023-06097-0.

## Background

Retained placenta, a condition where the placenta is not spontaneously expelled within 30 min after childbirth, is one of the obstetric complications [1, 2]. It can happen in up to 3.3% of births around the world, depending on the area [2–9]. The incidence of retained placenta is expected to rise due to increasing maternal age and risk factors such as previous cesarean delivery [10, 11]. It is the second leading cause of postpartum bleeding and can result in a mortality rate of up to 10% without prompt treatment [3, 11–15]. To address this issue effectively, we need a thorough and updated understanding of the disease profiles. However, recent data on the incidence and outcomes of retained placenta in the American population, particularly over the last decade, are limited.

Disparities by race and ethnicity in reproductive health outcomes have been observed, with non-Hispanic black and non-Hispanic American Indian/Alaska Native women experiencing higher pregnancy-related mortality ratios [16, 17]. It is clinically relevant to investigate whether similar disparities exist in the incidence and outcomes of retained placenta during childbirth [1]. To address this knowledge gap, our study uses a large, nationally representative US database to evaluate the recent incidence, healthcare utilization, and mortality of retained placenta in women following vaginal delivery. We also aim to determine whether these outcomes vary across racial and ethnic groups.

## Methods

### Study design and data source

We used data from the National Inpatient Sample (NIS), the largest all-payer database of hospitalized patients in the United States. The NIS, which is part of the Healthcare Cost and Utilization Project (HCUP) administered by the Agency for Healthcare Research and Quality (AHRQ), is a component of the National Inpatient Sample., covers more than 95% of the United States population, estimating over 35 million hospitalizations nationally. The NIS captures a stratified sample of 20% of discharges from all United States community hospitals starting from 2012. More information on the design of the NIS can be found on HCUP online resources (http://www.hcup-us.ahrq.gov). The analysis of the NIS sample uses completely de-identified data with no risk of loss of confidentiality. We completed a data user agreement with the AHRQ prior to using the NIS database. The institutional review board of West China Hospital, Sichuan University deemed the research project exempt from approval because it is a secondary analysis of publicly available, anonymized data.

### Study population

This analysis encompassed women aged 18 years or older who were admitted to hospitals for the purpose of childbirth from 2016 to 2019. The identification of delivery hospitalizations was conducted using the International Classification of Diseases, Tenth Revision, Clinical Modification (ICD-10-CM), which was previously validated through study [18]. These codes are detailed in Supplemental Table S1. We excluded those who had cesarean deliveries from the current study because they rarely experience retained placentas. We also excluded birthing persons with missing data on race and ethnicity.

### Study outcomes

This study primarily aimed to examine the occurrence of retained placenta after childbirth, with a focus on racial and ethnic differences. The exposure factor was race and ethnicity, categorized according to the United States Census Bureau's classification in the NIS database. This classification enabled us to identify potential disparities across racial and ethnic groups (White, Black, Hispanic, Asian and Pacific Islander, Native American, and others). We identified cases of retained placenta using validated ICD-10-CM codes O72.0 (hemorrhage associated with retained, trapped, or adhered placenta), O73.0 (retained placenta without hemorrhage), and O73.1 (retained placenta with hemorrhage) [19, 20]. We examined all available discharge diagnoses (primary or secondary) for vaginal delivery and retained placenta. The secondary outcomes included racial and ethnic disparities in hospital mortality and healthcare utilization, which involved the length of hospital stay and the total hospitalization costs over the study period.

### Patient-level and hospital characteristics

We gathered data on patient-level and hospital-level characteristics from the NIS database. Patient-level characteristics included age, comorbidities, admission type (elective or non-elective), primary payer information (Medicare, Medicaid, private, self-pay, and others), and household income quartile by resident zip code. We used the Elixhauser Comorbidity Index (ECI), as defined by HCUP Clinical Classification Software [21], to assess comorbidity burden. The hospital encompassed bed size, which was categorized into small, medium, and big. Additionally, the teaching status of the hospital was classified as either rural, urban non-teaching, or urban teaching. Lastly, the geographic region of the hospital was delineated into four categories: Northeast, Midwest, South, and West.

### Statistical analysis

We reported descriptive statistics as mean (standard deviation) for continuous variables and absolute values (percentages) for categorical variables, as appropriate. We expressed the rate of retained placenta as a percentage, or per 100,000 per delivery hospitalizations. We described racial and ethnic differences in patient-level and hospital-level characteristics. We modeled the trend in probability (percentage) over time for retained placenta using logistic regression, accounting for the complex survey design. Multivariable linear or logistic regression models were employed to examine the correlations between retained placenta and in-hospital mortality, length of stay, and hospitalization expenditures. We conducted these analyses across racial and ethnic categories. Due to the small number of birthing persons in subcategories like Native Americans and others, we carried out stratified analyses between White and non-White mothers. The multivariable regression models were adjusted for various factors including age, admission style, income in the patient's zip code, primary expected payer, comorbidity, hospital bed size, hospital teaching status, and hospital area. The findings of the regression analyses were provided in the form of odds ratios (ORs) accompanied with 95% confidence intervals (CIs). The analyses were conducted by including the sampling weights and stratified sample design of the NIS in order to derive estimates that are representative of the entire nation. A statistically significant level was determined by considering a 2-tailed *P* value of less than 0.001. The analyses were performed using Stata 17 software (StataCorp LLC., College Station, Texas).

## Results

The study analyzed data from 2,769,628 birthing persons who underwent vaginal deliveries in the United States between 2016 and 2019. This data corresponded to an estimated 13,848,131 hospitalizations nationwide after applying sampling weights. Among these hospitalizations, 108,035 weighted cases (0.78% [95% CI, 0.76%-0.80%]) were diagnosed with retained placenta. The racial and ethnic composition of the cohort was as follows: 52.8% were White, 20.6% were Hispanic, 14.9% were Black, and 6.3% were Asian. Less than 5.0% of the study population consisted of Native American or individuals from other racial/ethnic backgrounds. The mean age of the overall sample was 29 years. It is worth noting that Asian and Pacific Islander maternal patients were older compared to White maternal patients, while Native American, Black, and Hispanic mothers were younger than White mothers. Table 1 presents a comprehensive overview of the fundamental characteristics of the groups, categorized according to race/ethnicity. The Hispanic, Asian, Pacific Islander, and Black mothers were more likely to visit the emergency department, whereas White and Native American mothers were more likely to be admitted electively. Moreover, a higher proportion of Black, Hispanic, and Native American mothers belonged to lower median socioeconomic status groups. Additionally, Black, Hispanic, and Native American mothers were more likely to have Medicaid coverage, while White mothers were more likely to have private insurance. Asian and Pacific Islander mothers had a higher prevalence of comorbidities. Black mothers were less likely to be from hospitals in the West, while Native American birthing individuals were more likely to be from small hospitals and rural locations.

**Table 1:** Baseline patient-level and hospital characteristics of the study population by Race/Ethnicity (2016–2019)

Characteristic	Overall *n* = 13,848,131 (100%)	White *n* = 7,315,524 (52.8%)	Black *n* = 2,062,880 (14.9%)	Hispanic *n* = 2,851,338 (20.6%)	Asian and Pacific Islander *n* = 877,014 (6.3%)	Native American *n* = 101,480 (0.7%)	Others *n* = 639,895 (4.6%)
**Age, mean (SD), y**	29.1 (0.02)	29.4 (0.03)	27.8 (0.03)	28.4 (0.02)	31.5 (0.04)	27.7 (0.06)	29.5 (0.05)
**Age group, n (%)**							
18–24	3,284,163 (23.7)	1,516,038 (20.7)	672,910 (32.6)	845,519 (29.7)	76,225 (8.7)	32,505 (32.0)	140,965 (22.0)
25–34	4,058,922 (29.3)	2,171,093 (29.7)	630,100 (30.5)	827,510 (29.0)	217,750 (24.8)	31,345 (30.9)	181,125 (28.3)
35–44	3,997,258 (28.9)	2,304,968 (31.5)	456,655 (22.1)	687,185 (24.1)	337,365 (38.5)	23,945 (23.6)	187,140 (29.3)
≥ 45	2,507,789 (18.1)	1,323,424 (18.1)	303,215 (14.7)	491,125 (17.2)	245,675 (28.0)	13,685 (13.5)	130,665 (20.4)
**Year, n (%)**							
2016	3,494,141 (25.2)	1,860,423 (25.4)	511,465 (24.8)	713,849 (25.0)	221,745 (25.3)	26,120 (25.7)	160,540 (25.1)
2017	3,462,191 (25.0)	1,815,368 (24.8)	522,760 (25.3)	711,469 (25.0)	223,260 (25.5)	24,985 (24.6)	164,350 (25.7)
2018	3,460,769 (25.0)	1,827,409 (25.0)	510,195 (24.7)	717,535 (25.2)	217,220 (24.8)	24,850 (24.5)	163,560 (25.6)
2019	3,431,029 (24.8)	1,812,324 (24.8)	518,460 (25.1)	708,485 (24.8)	214,790 (24.5)	25,525 (25.2)	151,445 (23.7)
**Type of admission, n (%)**							
Non-elective	6,933,867 (50.2)	3,272,828 (44.8)	1,149,560 (55.8)	1,636,354 (57.5)	492,240 (56.2)	48,965 (48.3)	333,920 (52.3)
Elective	6,887,564 (49.8)	4,026,946 (55.2)	909,485 (44.2)	1,210,514 (42.5)	383,590 (43.8)	52,365 (51.7)	304,665 (47.7)
**Median household income for patient's ZIP Code, n (%)**							
≤ $43,999	3,861,223 (28.1)	1,550,408 (21.4)	1,004,945 (49.1)	1,001,060 (35.6)	105,720 (12.1)	39,535 (41.2)	159,555 (25.2)
$44,000–55,999	3,435,687 (25.0)	1,901,388 (26.2)	476,895 (23.3)	751,019 (26.7)	137,545 (15.8)	24,750 (25.8)	144,090 (22.7)
$56,000–73,999	3,394,883 (24.7)	1,977,818 (27.2)	356,140 (17.4)	666,940 (23.7)	217,910 (25.0)	18,660 (19.4)	157,415 (24.8)
≥ $74,000	3,027,638 (22.1)	1,828,869 (25.2)	209,275 (10.2)	394,060 (14.0)	409,620 (47.0)	13,075 (13.6)	172,740 (27.3)
**Expected primary payer, n (%)**							
Medicare	100,620 (0.7)	51,095 (0.7)	29,215 (1.4)	12,655 (0.4)	2,650 (0.3)	675 (0.7)	4,330 (0.7)
Medicaid	5,878,861 (42.5)	2,212,673 (30.3)	1,294,470 (62.8)	1,776,279 (62.4)	229,865 (26.2)	63,455 (62.8)	302,120 (47.2)
Private insurance	7,113,180 (51.4)	4,685,662 (64.1)	644,670 (31.3)	891,344 (31.3)	570,170 (65.0)	31,570 (31.3)	289,765 (45.3)
Self-pay	361,460 (2.6)	119,250 (1.6)	44,665 (2.2)	115,705 (4.1)	54,550 (6.2)	2,275 (2.3)	25,015 (3.9)
No charge	9,250 (0.1)	2,140 (0.0)	1,820 (0.1)	3,990 (0.1)	335 (0.0)	30 (0.0)	935 (0.1)
Other	369,885 (2.7)	235,715 (3.2)	46,205 (2.2)	48,695 (1.7)	19,025 (2.2)	2,980 (3.0)	17,265 (2.7)
**Elixhauser Comorbidity Index, n (%)**							
0	10,297,716 (74.3)	5,399,000 (73.8)	1,408,050 (68.3)	2,213,289 (77.6)	707,999 (80.7)	67,515 (66.5)	499,995 (78.1)
1	2,772,464 (20.0)	1,488,304 (20.3)	490,325 (23.8)	513,285 (18.0)	141,820 (16.2)	24,830 (24.5)	113,900 (17.8)
2	624,680 (4.5)	343,850 (4.7)	128,355 (6.2)	101,440 (3.6)	22,610 (2.6)	6,950 (6.8)	21,475 (3.4)
≥ 3	155,140 (1.1)	84,370 (1.2)	36,150 (1.8)	23,325 (0.8)	4,585 (0.5)	2,185 (2.2)	4,525 (0.7)
**Bedsize of hospital, n (%)**							
Small	2,601,325 (18.8)	1,490,001 (20.4)	350,715 (17.0)	486,459 (17.1)	144,045 (16.4)	22,810 (22.5)	107,295 (16.8)
Medium	4,228,524 (30.5)	2,170,397 (29.7)	648,485 (31.4)	895,349 (31.4)	262,440 (29.9)	27,100 (26.7)	224,755 (35.1)
Large	7,018,282 (50.7)	3,655,126 (50.0)	1,063,680 (51.6)	1,469,530 (51.5)	470,530 (53.7)	51,570 (50.8)	307,845 (48.1)
**Location/teaching status of hospital, n (%)**							
Rural	1,227,361 (8.9)	910,332 (12.4)	123,345 (6.0)	121,884 (4.3)	19,690 (2.2)	29,680 (29.2)	22,430 (3.5)
Urban nonteaching	2,907,618 (21.0)	1,611,223 (22.0)	318,905 (15.5)	639,980 (22.4)	182,095 (20.8)	16,250 (16.0)	139,165 (21.7)
Urban teaching	9,713,152 (70.1)	4,793,968 (65.5)	1,620,630 (78.6)	2,089,474 (73.3)	675,230 (77.0)	55,550 (54.7)	478,300 (74.7)
**Region of hospital, n (%)**							
Northeast	2,262,905 (16.3)	1,254,900 (17.2)	296,345 (14.4)	369,285 (13.0)	168,740 (19.2)	6,265 (6.2)	167,370 (26.2)
Midwest	2,806,597 (20.3)	1,953,358 (26.7)	411,495 (19.9)	232,965 (8.2)	110,165 (12.6)	20,480 (20.2)	78,135 (12.2)
South	5,512,178 (39.8)	2,736,384 (37.4)	1,179,675 (57.2)	1,094,250 (38.4)	192,535 (22.0)	28,015 (27.6)	281,320 (44.0)
West	3,266,450 (23.6)	1,370,882 (18.7)	175,365 (8.5)	1,154,839 (40.5)	405,574 (46.2)	46,720 (46.0)	113,070 (17.7)
**Retained placenta, n (per 100,000)**	108,035 (780)	60,794 (831)	14,065 (682)	19,845 (696)	7,490 (854)	1,455 (1,434)	4,385 (685)
**Mortality, n (per 100,000)**	815 (59)	335 (46)	180 (87)	160 (56)	80 (91)	20 (197)	40 (63)
**Length of stay, mean (SD), days**	2.6 (0.01)	2.6 (0.01)	2.9 (0.01)	2.6 (0.01)	2.7 (0.01)	2.6 (0.03)	2.7 (0.01)
**Total hospitalization cost, mean (SD), $**	5,936.1 (38.2)	5,499.6 (34.0)	6,285.3 (54.8)	6,495.6 (59.1)	6,806.2 (97.2)	5,112.5 (80.3)	6,284.9 (80.7)

Frequencies (%) in the columns may not sum to 100% because there might be missing data. Data weighted using sampling weights to achieve nationally representative estimates. Data presented as mean (standard deviation [SD]) or n (%)

**Fig. 1:**
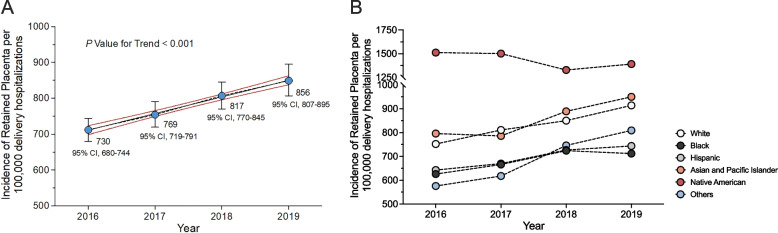
Incidence of retained placenta per 10,000 delivery hospitalizations in the United States, 2016–2019. **A** Incidence of retained placenta per 100,000 delivery hospitalizations among all study populations. Brackets around points are 95% confidence intervals. **B** Incidence of retained placenta per 100,000 delivery hospitalizations stratified by race/ ethnicity

Figure 1A illustrates the incidence of retained placenta per 100,000 delivery hospitalizations over the four-year survey period from 2016 to 2019. The data shows an increase in the incidence rate, with a statistically significant rise from 730 per 100,000 (0.73%) in 2016 to 856 per 100,000 (0.86%) in 2019 (*P* value for trend < 0.001). However, when subgroup analysis is performed based on race and ethnicity, the incidence of retained placenta per 100,000 among Native Americans remained stable or slightly decreased over the study period, while it significantly increased among other racial and ethnic groups (Fig. 1B). In contrast to a slight decline from 1.51% to 1.39% for Native Americans, the retained placenta rate increased from 0.63% in 2016 to 0.71% in 2019 for Black individuals, from 0.64% to 0.74% for the Hispanic population, and from 0.80% to 0.95% for Asians over the survey period (Fig. 1B).

The estimated incidence of retained placenta varied significantly by race and ethnicity, with Native Americans having the highest rate of 1,434 per 100,000 (1.43%) delivery hospitalizations, while Black and Hispanic women had lower rates of 682 and 696 per 100,000 delivery hospitalizations, respectively (Table 2). After adjusting for patient-level and hospital characteristics, Native American birthing had a higher likelihood of experiencing retained placenta (OR, 1.56; 95% CI, 1.35 to 1.81), whereas Black and Hispanic women were significantly less likely than White women to have retained placenta (OR, 0.92; 95% CI, 0.88 to 0.97 for Blacks and OR, 0.84; 95% CI, 0.80 to 0.89 for Hispanics). Table 2 details additional risk factors for retained placenta, including senior age, non-elective admission, higher ECI, and hospital region (all *P* < 0.001).

**Table 2:** Multivariate analysis of the association of patient-level and hospital characteristics with retained placenta

	**Incidence of retained placenta per 100,000 (95% CI)**	**Without retained placenta *N* = 13,740,096**	**With retained placenta *N* = 108,035**	**Adjusted OR (95% CI)**	***P*-Value**
**Race/Ethnicity**					
White	831 (810–852)	7,254,729 (52.8)	60,795 (56.3)	1 [Ref.]	
Black	682 (653–712)	2,048,815 (14.9)	14,065 (13.0)	0.92 (0.88–0.97)	0.001
Hispanic	696 (658–736)	2,831,493 (20.6)	19,845 (18.4)	0.84 (0.80–0.89)	< 0.001
Asian and Pacific Islander	854 (802–910)	869,524 (6.3)	7,490 (6.9)	0.89 (0.84–0.95)	< 0.001
Native American	1,434 (1,230–1,670)	100,025 (0.7)	1,455 (1.3)	1.56 (1.35–1.81)	< 0.001
Others	685 (638–736)	635,510 (4.6)	4,385 (4.1)	0.88 (0.81–0.94)	< 0.001
**Age group, y**					
18–24	607 (583–633)	3,264,213 (23.8)	19,950 (18.5)	1 [Ref.]	
25–34	689 (667–711)	4,030,952 (29.3)	27,970 (25.9)	1.10 (1.05–1.15)	< 0.001
35–44	857 (832–884)	3,962,983 (28.8)	34,275 (31.7)	1.34 (1.28–1.40)	< 0.001
45 or older	1,030 (997–1,065)	2,481,949 (18.1)	25,840 (23.9)	1.59 (1.52–1.67)	< 0.001
**Type of admission**					
Elective	718 (697–740)	6,838,109 (49.9)	49,455 (45.9)	1 [Ref.]	
Non-elective	841 (815–868)	6,875,572 (50.1)	58,295 (54.1)	1.18 (1.13–1.23)	< 0.001
**Median household income for patient's ZIP Code**					
First QT	687 (660–714)	3,834,708 (28.2)	26,515 (24.8)	1 [Ref.]	
Second QT	767 (742–792)	3,409,347 (25)	26,340 (24.6)	1.04 (0.99–1.08)	0.084
Third QT	819 (792–847)	3,367,073 (24.7)	27,810 (26.0)	1.05 (1.00–1.09)	0.035
Fourth QT	870 (837–904)	3,001,298 (22.0)	26,340 (24.6)	1.05 (0.99–1.10)	0.064
**Health insurance**					
Private insurance	831 (808–854)	7,054,080 (51.4)	59,100 (54.8)	1 [Ref.]	
Medicare	924 (794–1,075)	99,690 (0.7)	930 (0.9)	1.12 (0.95–1.31)	0.753
Medicaid	719 (695–743)	5,836,591 (42.5)	42,270 (39.2)	0.99 (0.96–1.03)	0.171
Self-pay	740 (673–814)	358,785 (2.6)	2,675 (2.5)	1.05 (0.95–1.15)	0.345
No charge	757 (440–1,299)	9,180 (0.1)	70 (0.1)	1.12 (0.65–1.93)	0.672
Other	779 (716–847)	367,005 (2.7)	2,880 (2.7)	1.02 (0.93–1.11)	0.680
**Elixhauser Comorbidity Index**					
0	719 (702–737)	10,221,782 (74.4)	74,065 (68.6)	1 [Ref.]	
1	911 (879–944)	2,747,214 (20.0)	25,250 (23.4)	1.22 (1.18–1.27)	< 0.001
2	1,074 (1,013–1,139)	617,970 (4.5)	6,710 (6.2)	1.40 (1.32–1.49)	< 0.001
≥ 3	1,296 (1,175–1,429)	153,130 (1.1)	2,010 (1.9)	1.64 (1.48–1.81)	< 0.001
**Bedsize of hospital**					
Large	818 (790–846)	6,960,882 (50.7)	57,400 (53.1)	1 [Ref.]	
Small	789 (755–825)	2,580,800 (18.8)	20,525 (19.0)	0.99 (0.94–1.05)	0.760
Medium	712 (683–743)	4,198,414 (30.6)	30,110 (27.9)	0.94 (0.89–0.99)	0.032
**Location/teaching status of hospital**					
Urban teaching	816 (793–840)	9,633,882 (70.1)	79,270 (73.4)	1 [Ref.]	
Rural	783 (742–825)	1,217,756 (8.9)	9,605 (8.9)	1.03 (0.96–1.10)	0.398
Urban nonteaching	659 (631–688)	2,888,458 (21.0)	19,160 (17.7)	0.84 (0.80–0.89)	< 0.001
**Region of hospital**					
South	544 (519–571)	5,482,178 (39.9)	30,000 (27.8)	1 [Ref.]	
Northeast	795 (755–837)	2,244,920 (16.3)	17,985 (16.6)	1.35 (1.25–1.45)	< 0.001
Midwest	966 (925–1,009)	2,779,487 (20.2)	27,110 (25.1)	1.68 (1.58–1.79)	< 0.001
West	1,008 (967–1,051)	3,233,510 (23.5)	32,940 (30.5)	1.79 (1.67–1.92)	< 0.001

Frequencies (%) in the columns may not sum to 100% because there might be missing data. Data weighted using sampling weights to achieve nationally representative estimates. Multivariable model adjusted for age, race/ethnicity, type of admission, income in the patient’s zip code, expected primary payer, Elixhauser Comorbidity Index, bed size of the hospital, location/teaching status of hospital, and region of hospital except for the variable itself

*OR* odds ratio, *CI* confidence interval

Moreover, an in-depth analysis of the association between retained placenta and secondary outcomes was conducted using the survey data (Table 3). Deliveries with retained placenta were found to increase the healthcare burden and the risk of death, particularly among non-White women. Adjusting for patient-level and hospital characteristics, deliveries with retained placenta were associated with longer hospital stays (0.18 days, 95% CI, 0.13 to 0.23) and higher total costs ($907.1, 95% CI, 742.9 to 1,071.3). These effects were more pronounced among non-White women (total costs: $1,128.0, 95% CI, 889.9 to 1,366.1) in subgroup analysis. Notably, birthing persons with retained placenta were over three times more likely to die than those without retained placenta (OR, 3.48; 95% CI, 1.53 to 7.91). However, the increased mortality risk was observed only in non-White women in subgroup analysis (OR, 4.81; 95% CI, 1.95 to 11.87).

**Table 3:** Logistic regression analysis for the association between the retained placenta and secondary outcomes

**Outcome/group**	**Crude model**		**Adjusted model**	
	**OR / β (95% CI)**	***P*-Value**	**OR / β (95% CI)**	***P*-Value**
**Inpatient mortality**				
Overall	4.86 (2.16–10.96)	< 0.001	3.48 (1.53–7.91)	< 0.001
Race/ethnicity				
White	1.82 (0.25–13.04)	0.557	1.46 (0.20–10.85)	0.711
Non-White	7.55 (3.06–18.63)	< 0.001	4.81 (1.95–11.87)	0.001
**Length of stay, days**				
Overall	0.22 (0.18–0.27)	< 0.001	0.18 (0.13–0.23)	< 0.001
Race/ethnicity				
White	0.24 (0.18–0.31)	< 0.001	0.20 (0.13–0.26)	< 0.001
Non-White	0.21 (0.14–0.28)	< 0.001	0.15 (0.08–0.22)	< 0.001
**Total hospitalization cost, $**				
Overall	1,284.4 (1,133.3–1,435.4)	< 0.001	973.1 (829.4–1,116.9)	< 0.001
Race/ethnicity				
White	1,186.5 (1,018.0–1,355.0)	< 0.001	907.1 (742.9–1,071.3)	< 0.001
Non-White	1,485.2 (1,234.1–1,736.4)	< 0.001	1,128.0 (889.9–1,366.1)	< 0.001

Multivariable model adjusted for age, race/ethnicity, type of admission, income in the patient’s zip code, expected primary payer, Elixhauser Comorbidity Index, bed size of the hospital, location/teaching status of the hospital, and region of the hospital

*OR* odds ratio, *CI* confidence interval

## Discussion

This comprehensive study, based on a large and racially diverse United States population, provides updated estimates on the incidence and outcomes of retained placenta during vaginal deliveries. Our findings reveal that retained placenta occurs in 1 out of every 128 delivery hospitalizations, signifying a significant health and economic impact in the United States. Furthermore, we identified substantial racial and ethnic disparities in the incidence, healthcare utilization, and outcomes of retained placenta during delivery hospitalizations.

Our study aligns with previous research, estimating the incidence rate of retained placenta at 0.78% (1 in 128) among delivery hospitalizations, which is consistent with previous reports of an incidence ranging from 0.1% to 3.3% [2–4, 6, 9]. However, it is important to note that the reported incidence rates of retained placenta vary widely across studies, with lower incidence rates often reported in less industrialized nations [4]. The reasons for this variation are complex and multifactorial, potentially related to diverse epidemiological or delivery-related risk factors such as previous cesarean delivery, maternal age, and oxytocin use, as well as different practice settings worldwide [2, 4, 7–9, 11–13]. We observed an increase in the incidence of retained placenta over the study period, the reasons for which are largely unknown and warrant further investigation. Some studies suggest factors such as increased cesarean delivery rate and maternal age may contribute to the elevated incidence of retained placenta [6, 20, 22], but these factors remain controversial.

Although the adverse maternal health outcomes affected by race and ethnicity have been extensively reported [16, 23–25], very few studies investigate the disparities in retained placenta. In an initial study based on a small sample of the American population, Coviello et al. found that the non-Hispanic Black race was associated with a decreased risk of retained placenta compared with the non-Hispanic White race. In contrast, no association with other races was found [6]. Our study fills the data gap left by the above research by showing more detailed information regarding the disparities of racial and ethnic groups in the incidence of retained placenta among delivery hospitalizations in the United States. We showed that the retained placenta rates by race and ethnicity differed significantly more than previously reported. In addition to Blacks, as constant with Coviello et al. [6], compared to White mothers, Hispanic and Asian, and Pacific Islander birthing persons were significantly less likely to experience retained placenta than White women, while Native American birthing persons are more likely to experience retained placenta. Although the causes of these disparities are not yet elucidated, we assume that they may be connected to social determinants and delivery-related risk factors. For example, we recorded that the proportion of Native American birthing people (nearly 30%) located in rural settings, where medical resources are relatively inadequate, is substantially higher than other races/ethnicities (less than 13%). Previous study suggested that mothers living in rural areas are less likely to utilize skilled birthing attendants as compared to their counterparts living in urban areas [26]. Consequently, the lack of skilled birthing attendants may contribute to these disparities by delaying early detection and management of complications like retained placenta among Native American birthing persons during the birth process. In addition, delivery-related risk factors, such as previous cesarean delivery, maternal age, and oxytocin use, were thought to be associated with variation in the incidence rates of retained placenta that may also be related to racial disparities in retained placenta due to different cultural and practice setting. However, there is currently no direct evidence to support these hypotheses. Nevertheless, our findings highlighting clinical care or health policy efforts aimed at reducing the disparate and increasing incidence of retained placenta may need to be given priority to those Native American birthing population who living in rural areas. Further studies are encouraged to explore the intrinsic mechanism for this observation, with opportunities for potential management strategy.

Our estimate of a 2.8% in-hospital mortality rate among deliveries with retained placenta aligns with previous estimates ranging from 3% to 9% [3]. Our results also showed that birthing persons with retained placenta were nearly 3.5 times more likely to die than those without retained placenta, underscoring the importance of effective management of retained placenta in obstetric care. We also found that deliveries with retained placenta significantly increased healthcare utilization, as measured by length of stay and hospitalization costs. Notably, significant racial and ethnic differences were observed in the healthcare burden and outcomes of retained placenta during labor and delivery hospitalization. For instance, in our subgroup analyses, non-White women were associated with higher in-hospital mortality and total hospitalization costs compared to White women. These findings highlight the need for attentive pregnancy management, which may contribute to preconception counseling and improved maternal outcomes.

Our study has several strengths,which utilizes the NIS—the largest public dataset for hospitalizations—offers several strengths. The large sample size of the NIS ensures our findings are representative of the entire United States population. Besides, our study examines the occurrence of retained placenta among pregnant women during delivery from 2016 to 2019, making our findings pertinent to current obstetric practices. Crucially, our data offer unique insights into how racial and ethnic disparities in the incidence, healthcare burden, and outcomes of retained placenta, based on extensive, nationwide, racially diverse data.

However, our study also presents several limitations. Firstly, the identification of these events relies heavily on the accuracy of administrative data coding. We mitigated this as much as possible by using several validated measures. For instance, we validated the diagnosis rate of retained placenta with the frequency distributions of the diagnoses and procedure codes from 2016 to 2019 provided by the HCUP NIS. Secondly, due to the nature of the NIS database, we cannot track patients longitudinally over time after discharge. This means we could not account for post-discharge events of retained placenta, limiting our ability to estimate the incidence rate of post-discharge retained placenta other than those occurring at the time of delivery hospitalization. This limitation is similar to the rate of out-of-hospital births, which is relatively low in the United States [27]. Thirdly, with a large sample size, even small differences between the groups analyzed could be statistically significant, but their practical or clinical significance may be questionable. For instance, delivery with retained placenta is associated with a longer hospital stay of 0.18 days, which may not be clinically significant. Lastly, due to the small number of women in subcategories like Native Americans and others, we only conducted stratified analyses between White and non-White delivery women, which could limit the insights into racial/ethnic disparities in study outcomes. Future analyses should, whenever possible, involve each subgroup to improve understanding in this field. Addressing these concerns will be essential in future studies.

## Conclusions

This national survey revealed that the incidence of retained placenta was 0.78% (1 in 128) among among people undergoing vaginal delivery. Over the investigated time period, the incidence of retained placenta in the United States to be trending upward. The study also documented significant racial disparities in the incidence and outcomes of retained placenta during hospitalization for childbirth. The condition is associated with higher in-hospital mortality, more extended hospital stays, and higher total hospitalization costs among pregnant women at delivery, thus representing a substantial health and economic burden in the United States. These findings provide evidence-based data on the profile and inequality of maternity care for retained placentas. It is suggested that specific interventions like well-equipping skilled birth attendants and improving access to healthcare efforts aimed at reducing the disparity and increasing the incidence of retained placenta may be given priority, especially for Native American birthing women living in rural areas.

### Supplementary Information

**Additional file 1:** **Supplemental Table 1. **Study Definitions by ICD-10-CM Codes.

## Data Availability

The data supporting the findings of this study are available from the HCUP, which is the Nation most comprehensive source of hospital care data, including information on inpatient stays, ambulatory surgery and services visits, and emergency department encounters. It is important to note that there are certain limitations regarding the accessibility of the aforementioned data. These data were utilized in the present study under a licensing agreement, hence rendering them inaccessible to the general public. However, data can be obtained from the associated author upon a reasonable request and with the approval of HCUP.

## Abbreviations

CI: Confidence interval
ECI: Elixhauser Comorbidity Index
HCUP: Healthcare Cost and Utilization Project
ICD-10-CM: International Classification of Diseases, Tenth Revision, Clinical Modification
NIS: National inpatient sample
OR: Odds ratio
