# Supplementary material for: Racial and ethnic disparities in the incidence, healthcare utilization, and outcomes of retained placenta among delivery hospitalizations in the United States, 2016–2019
Source: BMC Pregnancy Childbirth. 2023 Nov 11;23:783. doi: 10.1186/s12884-023-06097-0 (PMC10638702; doi:10.1186/s12884-023-06097-0)
Supplement: Supplementary file 1 — Additional file 1: Supplemental Table 1. Study Definitions by ICD-10-CM Codes. [file 12884_2023_6097_MOESM1_ESM.docx]

| **Supplemental Table 1. Study Definitions by ICD-10-CM Codes** |
| --- |
|  |
| **ICD-10 Code descriptions for retained placenta** |
| Comment: Retained placenta is of the interest outcome. Clinical retained placenta defined as the absence of complete placental release within 30 minutes of delivery. Here, retained placenta was defined according to the International Classification of Diseases, Tenth Revision, Clinical Modification (ICD-10-CM). |
| 072.0 - Third-stage hemorrhage (Inclusion terms: Hemorrhage associated with retained, trapped or adherent placenta; Retained placenta NOS) |
| 073.0 - Retained placenta without hemorrhage (Inclusion terms: Adherent placenta, without hemorrhage; Trapped placenta without hemorrhage) |
| 073.1 - Retained portions of placenta and membranes, without hemorrhage |
|  |
| **ICD-10 Code descriptions for vaginal delivery** |
| 060.1x - Preterm labor with preterm delivery |
| 060.2x - Term delivery with preterm labor |
| 063.0 - Prolonged first stage (of labor) |
| 063.1 - Prolonged second stage (of labor) |
| 063.2 - Delayed delivery of second twin, triplet, etc. |
| 063.9 - Long labor, unspecified |
|  |
| 064.0 - Obstructed labor due to incomplete rotation of fetal head |
| 064.1 - Obstructed labor due to breech presentation |
| 064.2 - Obstructed labor due to face presentation |
| 064.3 - Obstructed labor due to brow presentation |
| 064.4 - Obstructed labor due to shoulder presentation |
| 064.5 - Obstructed labor due to compound presentation |
| 064.8 - Obstructed labor due to other malposition and malpresentation |
| 064.9 - Obstructed labor due to malposition and malpresentation, unspecified |
|  |
| 066.0 - Obstructed labor due to shoulder dystocia |
| 066.1 - Obstructed labor due to locked twins |
| 066.2 - Obstructed labor due to unusually large fetus |
| 066.3 - Obstructed labor due to other abnormalities of fetus |
| 066.5 - Attempted application of vacuum extractor and forceps |
| 066.6 - Obstructed labor due to other multiple fetuses |
| 066.8 - Other specified obstructed labor |
| 066.9 - Obstructed labor, unspecified |
|  |
| 069.0 - Labor and delivery complicated by prolapse of cord |
| 069.1 - Labor and delivery complicated by cord around neck, with compression |
| 069.2 - Labor and delivery complicated by other cord entanglement, with compression |
| 069.3 - Labor and delivery complicated by short cord |
| 069.4 - Labor and delivery complicated by vasa previa |
| 069.5 - Labor and delivery complicated by vascular lesion of cord |
| 069.8 - Labor and delivery complicated by other cord complications |
| 069.9 - Labor and delivery complicated by cord complication, unspecified |
|  |
| 070.0 - First degree perineal laceration during delivery |
| 070.1 - Second degree perineal laceration during delivery |
| 070.2 - Third degree perineal laceration during delivery |
| 070.3 - Fourth degree perineal laceration during delivery |
| 070.4 - Anal sphincter tear complicating delivery, not associated with third degree laceration |
| 070.9 - Perineal laceration during delivery, unspecified |
|  |
| 074.0 - Aspiration pneumonitis due to anesthesia during labor and delivery |
| 074.1 - Other pulmonary complications of anesthesia during labor and delivery |
| 074.2 - Cardiac complications of anesthesia during labor and delivery |
| 074.3 - Central nervous system complications of anesthesia during labor and delivery |
| 074.4 - Toxic reaction to local anesthesia during labor and delivery |
| 074.5 - Spinal and epidural anesthesia-induced headache during labor and delivery |
| 074.6 - Other complications of spinal and epidural anesthesia during labor and delivery |
| 074.7 - Failed or difficult intubation for anesthesia during labor and delivery |
| 074.8 - Other complications of anesthesia during labor and delivery |
| 074.9 - Complication of anesthesia during labor and delivery, unspecified |
|  |
| 075.0 - Maternal distress during labor and delivery |
| 075.1 - Shock during or following labor and delivery |
| 075.2 - Pyrexia during labor, not elsewhere classified |
| 075.3 - Other infection during labor |
| 075.4 - Other complications of obstetric surgery and procedures |
| 075.5 - Delayed delivery after artificial rupture of membranes |
| 075.8 - Other specified complications of labor and delivery |
| 075.9 - Complication of labor and delivery, unspecified |
|  |
| 077.0 - Labor and delivery complicated by meconium in amniotic fluid |
| 077.1 - Fetal stress in labor or delivery due to drug administration |
| 077.8 - Labor and delivery complicated by other evidence of fetal stress |
| 077.9 - Labor and delivery complicated by fetal stress, unspecified |
|  |
| 080 - Encounter for full-term uncomplicated delivery |
|  |
| Z37.0 - Single live birth |
| Z37.1 - Single stillbirth |
| Z37.2 - Twins, both liveborn |
| 237.3 - Twins, one liveborn and one stillborn |
| 237.4 - Twins, both stillborn |
| 237.5x - Other multiple births, all liveborn |
| 737.6x - Other multiple births, some liveborn |
| 237.7 - Other multiple births, all stillborn |
| Z37.9 - Outcome of delivery, unspecified |
|  |
| Z38.0x - Single liveborn infant, born in hospital |
| Z38.1 - Single liveborn infant, born outside hospital |
| Z38.2 - Single liveborn infant, unspecified as to place of birth |
| Z38.3x - Twin liveborn infant, born in hospital |
| Z38.4 - Twin liveborn infant, born outside hospital |
| Z38.5 - Twin liveborn infant, unspecified as to place of birth |
| Z38.6x - Other multiple liveborn infant, born in hospital |
| Z38.7 - Other multiple liveborn infant, born outside hospital |
| Z38.8 - Other multiple liveborn infant, unspecified as to place of birth |
|  |
| Z39.0 - Encounter for care and examination of mother immediately after delivery |
